# Supplementary material for: HIVprotI: an integrated web based platform for prediction and design of HIV proteins inhibitors
Source: J Cheminform. 2018 Mar 9;10:12. doi: 10.1186/s13321-018-0266-y (PMC5845081; doi:10.1186/s13321-018-0266-y)
Supplement: Supplementary file 2 — Additional file 2. Source code of HIVProtI web server. [file 13321_2018_266_MOESM2_ESM.zip › HIVprotI_Source-code/hivprotI/weka-3-6-6/doc/weka/classifiers/evaluation/MarginCurve.html]

MarginCurve


---


|  |  |  |
| --- | --- | --- |
| **Overview** \| **Package** \| **Class** \| **Tree** \| **Deprecated** \| **Index** \| **Help** \| **Weka's home** | |  |
| **PREV CLASS**   **NEXT CLASS** | **FRAMES**    **NO FRAMES**     **All Classes** |  |
| SUMMARY: NESTED \| FIELD \| CONSTR \| METHOD | DETAIL: FIELD \| CONSTR \| METHOD |  |


---


## weka.classifiers.evaluation Class MarginCurve

```
java.lang.Object
  weka.classifiers.evaluation.MarginCurve
```

**All Implemented Interfaces:**: RevisionHandler

---

``` public class MarginCurve extends java.lang.Object implements RevisionHandler ```

Generates points illustrating the prediction margin. The margin is defined
as the difference between the probability predicted for the actual class and
the highest probability predicted for the other classes. One hypothesis
as to the good performance of boosting algorithms is that they increaes the
margins on the training data and this gives better performance on test data.

**Version:**
:   $Revision: 1.11 $

**Author:**
:   Len Trigg (len@reeltwo.com)

---

**Constructor Summary**

`MarginCurve()`


| **Method Summary** | |
| --- | --- |
| `Instances` | `getCurve(FastVector predictions)`             Calculates the cumulative margin distribution for the set of predictions, returning the result as a set of Instances. |
| `java.lang.String` | `getRevision()`             Returns the revision string. |
| `static void` | `main(java.lang.String[] args)`             Tests the MarginCurve generation from the command line. |

**Methods inherited from class java.lang.Object**

`equals, getClass, hashCode, notify, notifyAll, toString, wait, wait, wait`

**Constructor Detail**

### MarginCurve

```
public MarginCurve()
```


**Method Detail**

### getCurve

```
public Instances getCurve(FastVector predictions)
```

:   Calculates the cumulative margin distribution for the set of
    predictions, returning the result as a set of Instances. The
    structure of these Instances is as follows:

    - **Margin** contains the margin value (which should be plotted
      as an x-coordinate)- **Current** contains the count of instances with the current
        margin (plot as y axis)- **Cumulative** contains the count of instances with margin
          less than or equal to the current margin (plot as y axis)

    :   **Returns:**: datapoints as a set of instances, null if no predictions have been made.

---


### getRevision

```
public java.lang.String getRevision()
```

:   Returns the revision string.

    :   **Specified by:**: `getRevision` in interface `RevisionHandler`
    :   **Returns:**: the revision

---


### main

```
public static void main(java.lang.String[] args)
```

:   Tests the MarginCurve generation from the command line.
    The classifier is currently hardcoded. Pipe in an arff file.

    :   **Parameters:**: `args` - currently ignored


---


|  |  |  |
| --- | --- | --- |
| **Overview** \| **Package** \| **Class** \| **Tree** \| **Deprecated** \| **Index** \| **Help** \| **Weka's home** | |  |
| **PREV CLASS**   **NEXT CLASS** | **FRAMES**    **NO FRAMES**     **All Classes** |  |
| SUMMARY: NESTED \| FIELD \| CONSTR \| METHOD | DETAIL: FIELD \| CONSTR \| METHOD |  |


---
